# Adult-onset neuronal intranuclear inclusion disease, with both stroke-like onset and encephalitic attacks: a case report

**DOI:** 10.1186/s12883-021-02164-1

**Published:** 2021-03-31

**Authors:** Ying Huang, Ge Jin, Qun-ling Zhan, Yun Tian, Lu Shen

**Affiliations:** 1 Department of Neurology, Chongqing Renji Hospital, University of Chinese Academy of Sciences, Chongqing, 400062 China; 2 Department of Neurology, Xiangya Hospital, Central South University, Changsha, 410008 Hunan China

**Keywords:** Acute cerebral infarction, Encephalitic attacks, Neuronal intranuclear inclusion disease, Magnetic resonance imaging, Skin biopsy, p62/ubiquitin staining, Genetic testing

## Abstract

**Background:**

Neuronal intranuclear inclusion disease (NIID) is a neurodegenerative disease, the clinical manifestations of which are complex and easily misdiagnosed. NIID clinical characteristics are varied, affecting the central and peripheral nervous systems and autonomic nerves. In this study, we present an NIID case with both stroke-like onset and encephalitic attacks, which is a rare case report.

**Case presentation:**

A 68-year-old Chinese female presented with sudden aphasia and limb hemiplegia as the first symptoms, as well as fever, cognitive impairment and mental irritability from encephalitic attacks. During hospitalization, a brain magnetic resonance imaging (MRI) examination detected high signal intensity from diffusion-weighted imaging (DWI) of the bilateral frontal grey matter-white matter junction. Electrophysiological tests revealed the main site of injury was at the myelin sheath in the motor nerves. A skin biopsy revealed eosinophilic spherical inclusion bodies in the nuclei of small sweat gland cells, fibroblasts and fat cells, whilst immunohistochemistry revealed that p62 and ubiquitin antibodies were positive. From genetic analyses, the patient was not a carrier of the fragile X mental retardation 1 (FMR1) permutation, but repeated GGC sequences in the *NOTCH2NLC* gene confirmed an NIID diagnosis. Through antipsychotic and nutritional support therapy, the patient’s symptoms were completely relieved within 3 weeks.

**Conclusions:**

This report of an NIID case with both stroke-like onset and encephalitic attacks provides new information for NIID diagnoses, and a comprehensive classification of clinical characteristics.

## Background

Neuronal intranuclear inclusion disease (NIID) is a chronic progressive neurodegenerative disorder, characterized by eosinophilic transparent inclusions in the central and peripheral nervous systems, and internal organs [1, 2]. Currently, the etiology and pathogenesis of NIID remains unclear. The pathogenesis of NIID is unknown. Immunohistochemically, intranuclear inclusions are positive for ubiquitin and ubiquitin related proteins, including p62, SUMO1, FUS, MYO6, and OPTN-C, suggesting the ubiquitin-proteasome pathway in the nucleus plays a role in NIID [3–7]. In addition, GGC repeat expansions in the 5′ untranslated region (5′ UTR) of the *NOTCH2NLC* (Notch 2 N-terminal like C) gene are also associated with NIID mechanisms [8–11].

Similarly, NIID clinical characteristics are varied, affecting the central and peripheral nervous systems and autonomic nerves. Sporadic NIID emerges between 51 and 76 years of age [12]. Due to this variability in clinical symptoms, NIID is considered a heterogeneous disease [13]. Patients frequently manifest with dementia (94.7%), muscle weakness (27%), sensory disturbances (28.6%), autonomic nerve dysfunction, ataxia, epilepsy, paroxysmal disturbances in consciousness (39.5%) and Parkinsonism [14]. In recent years, NIID has been diagnosed by skin biopsy, magnetic resonance imaging (MRI) and genetic testing.

We report adult-onset NIID in a patient who manifested with both aphasia and limb hemiplegia, as the first symptoms. During hospitalization, she also developed fever, reversible cognitive impairment and mental irritability from encephalitic attacks. This case report describes a rare clinical manifestation of NIID, which may be helpful in updating our understanding of NIID clinical characteristics for clinicians.

## Case presentation

A 68-year-old, right handed Chinese female of Han nationality, endured 3 years of untreated hypertension. She denied a history of smoking and diabetes. She had no family history of NIID or any other neurodegenerative disease. She was admitted to hospital, complaining of sudden aphasia and right limb weakness in the previous 4 h. In the past 7 years, the patient had recurrent aphasia and two episodes of limb hemiparesis, and was diagnosed with “transient ischemic attack” in 2012 and 2018. During hospitalization in 2012, she showed a sudden weakness in the right limb. This lasted 2 h and recovered without treatment. In 2018, the patient showed sudden aphasia and weakness in the right limb, which relived itself in 3 h. During these hospitalizations, the patient developed no mental abnormalities, but did have a fever > 38 °C. Each time, she was given anti-platelet aggregation therapy and supportive treatment such as vitamin B and C supplements. For most of the time, the patient looked after herself; she communicated well and was able to walk normally, however, in the recent 6 months, she suffered with memory loss and had issues with counting.

Upon admission to hospital, a physical examination revealed a temperature of 36.6 °C, a pulse of 90 beats/min, a breath rate of 20 times/min, and a blood pressure of 196/106 mmHg. The patient’s consciousness was normal, but she was unable to cooperate during the examination, and she also had mixed aphasia. She also had central facial and tongue paralysis on her left side. Her right limb muscle strength was 4.5/5.0. The tendon reflex of her extremities was active, meningeal irritation was negative, and the right Babinski and Chaddock signs were positive. The patient was unable to cooperate during sensory system and ataxia examinations.

On the 3rd day after admission, the patient developed new symptoms: fever and an exacerbation of right limb weakness. A physical examination revealed a temperature of 38.8 °C, and a motor examination revealed a right limb muscle strength of 3/5. Her meningeal irritation and right Babinski and Chaddock signs changed to negative. On the 5th day after admission, the patient had mental irritability and cognitive impairment. On the 20th day after admission, without any treatment which could have resulted in cognitive improvement, the patient’s limb muscle strength returned to normal, she had no mental abnormalities, but her memory and counting skills were still poor.

**Fig. 1:**
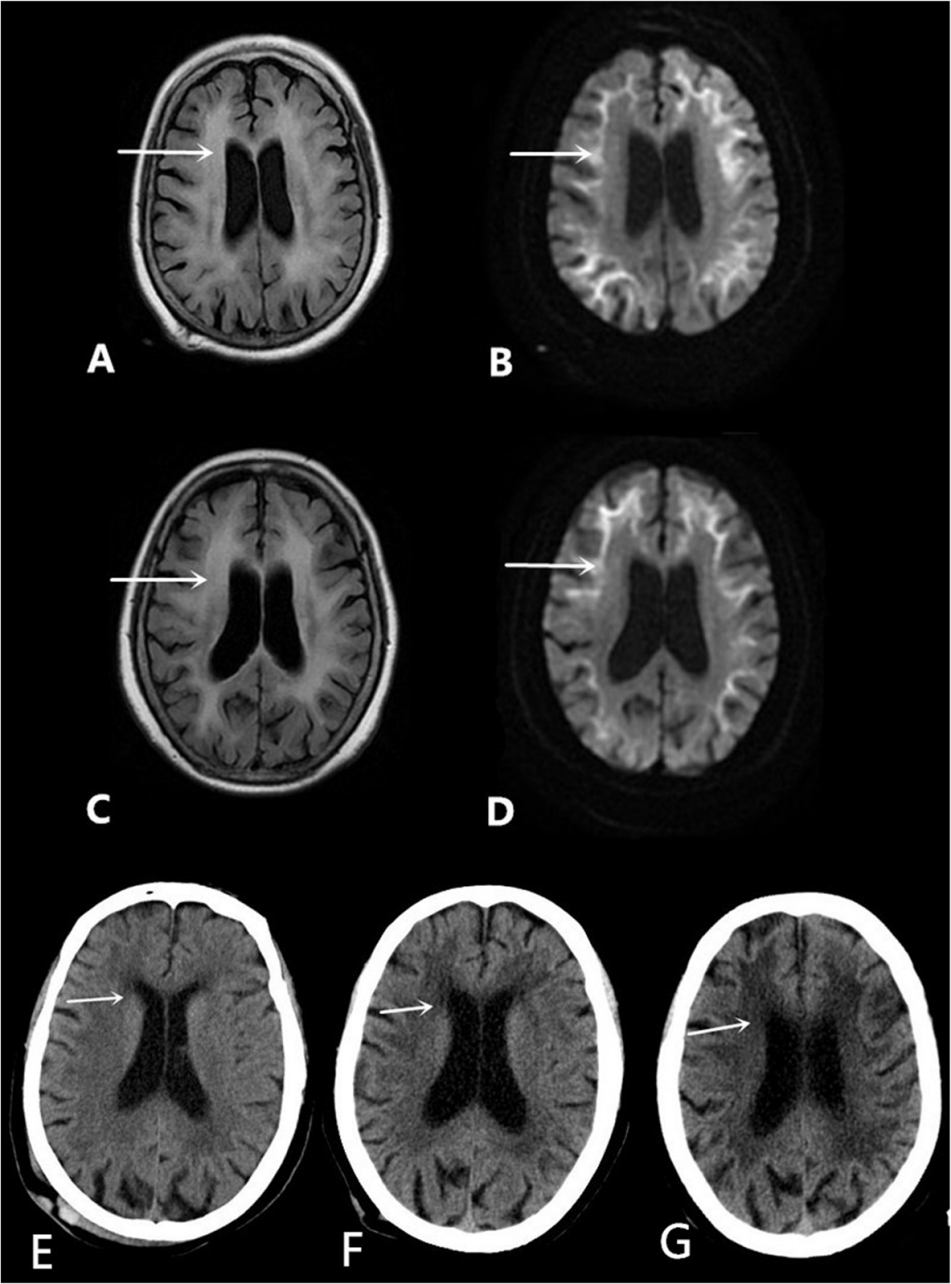
MRI scans on the 5th day after admission: **a** The T2/FLAIR sequence: the arrow refers to abnormal signals in the white matter region of the brain, **b** DWI imaging: the arrow refers to high intensity areas in the cerebral white matter and grey matter-white matter junction; the MRI was conducted on the 20th day after admission: **c** The T2/FLAIR sequence: the arrow refers to abnormal signals in the white matter region of the brain, **d** DWI imaging: the arrow refers to high intensity areas in the cerebral white matter and grey matter-white matter junction. Cranial computed tomography scans showed a low-density lesion in the white matter, area-brain atrophy, and leucoencephalopathy: **e** in 2012, **f** in 2018, and **g** this hospitalization

Computed tomography angiography (CTA) revealed no intracranial aortic stenosis or occlusion. On the 5th day after admission, a T2/Flair sequence of a skull magnetic resonance imaging (MRI) scan revealed abnormal signals in the white matter region of the brain, and high signal intensity in the bilateral frontal grey matter-white matter junction, by diffusion-weighted imaging (DWI) (Fig. 1a, c). The patient had a second MRI scan on the 20th day after admission, but results from both scans were not significantly different (Fig. 1b, d). During the previous two hospitalizations, due to various reasons, the patient failed to complete an MRI and electroencephalogram (EEG), but cranial computed tomography (CT) scans revealed a low-density lesion in the white matter, area-brain atrophy, and leucoencephalopathy. During each hospitalization, these symptoms worsened (Fig. 1e, f, g).

Routine blood tests revealed the patient’s leukocyte count was 12.29 × 10^9^/L (the normal range is 4.0–10.0 × 10^9^/L), and her neutrophil percentage was 89.4% (the normal range is 50–70%). Her C-reactive protein and procalcitonin levels were normal. A cerebrospinal fluid lumbar puncture examination revealed that pressure was 160 mmH2O.The number of cells in the CSF was 4.0 × 10^6^/L (the normal range is 4.0–10.0 × 10^9^/L), and a cytological examination of the cerebrospinal fluid revealed 50% lymphocytes and 50% monocytes. Biochemical tests revealed her glucose level was 3.8 mmol/L, total protein was 0.75 g/L (the normal range is 0.15–0.4 g/L), and chloride was 129.9 mmol/L (the normal range is 120-130 mmol/L).

On the 20th day after admission, the patient’s serial electroencephalogram (EEGs) demonstrated a low amplitude θ wave (5–7 Hz), and a few α rhythms (8–9 Hz), but no abnormal brain waves were observed. Her skin sympathetic reflexes revealed that the amplitude of her right upper limb (194.1 μV) was lower than the left (497.2 μV). Electrophysiological tests revealed that motor nerve conduction velocity of the left median nerve (47 m/s), right median nerve (49 m/s), left ulnar nerve (47 m/s), left common peroneal nerve (41 m/s) and right common peroneal nerve (40 m/s) were slow. The sensory conduction velocities of bilateral median, ulnar, and sural nerves were normal. The following scales were completed: The mini-mental state examination (MMSE) scale was 24/30, the Montreal cognitive assessment scale (MoCA) was 21/30, the frontal lobe function assessment (FAB) was 17, and the activity of daily living scale (ADL) was 21.

**Fig. 2:**
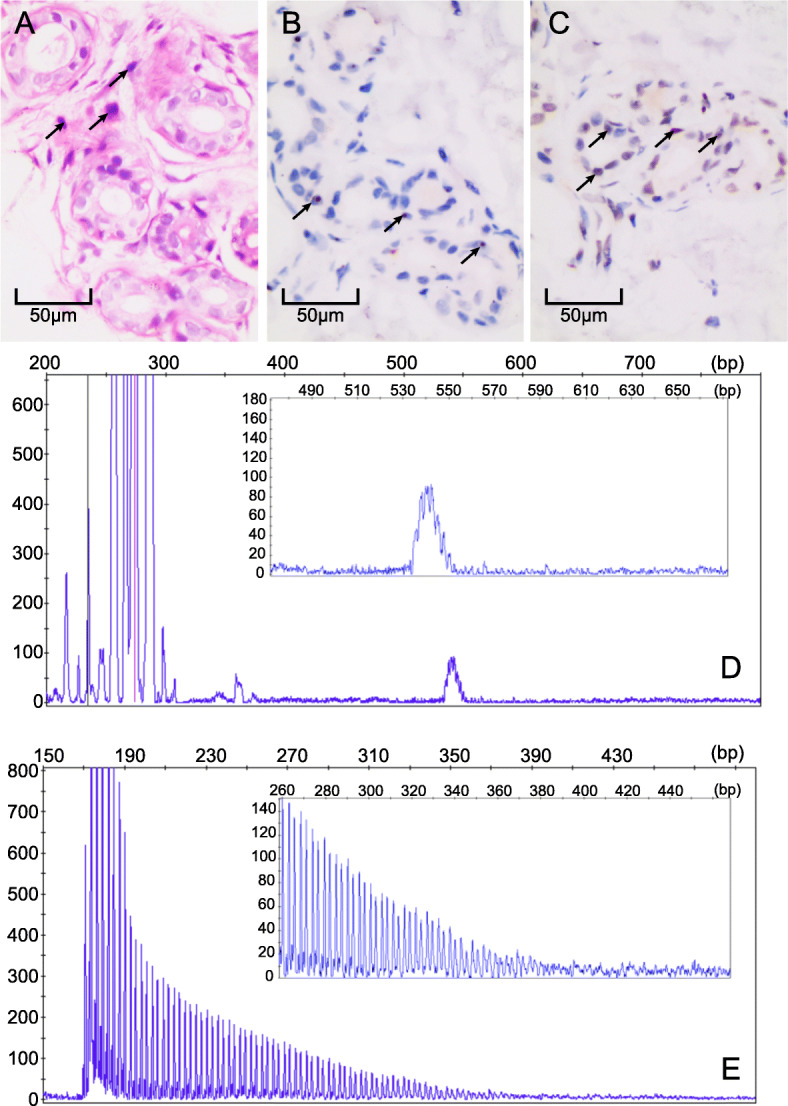
Skin biopsy samples underwent immunohistochemical staining: **a** hematoxylin-eosin staining (× 200 magnification), the arrows refer to eosinophilic spherical inclusion bodies in the nuclei of epithelial cells in small sweat gland cells; **b** anti-p62 immunohistochemical staining (arrows, × 200 magnification) and **c** anti-ubiquitin immunohistochemical staining (arrows, × 200 magnification). **d** Electropherograms showing GC-rich regions using PCR and repeat-primed PCR assays. **e** GC-rich PCR (GC-PCR) indicated the patient had 102 GGC repeats in the 5’ UTR of NOTCH2NLC

A skin biopsy was performed under light microscopy. Hematoxylin-eosin staining revealed eosinophilic spherical inclusion bodies in the nuclei of epithelial cells in small sweat gland cells (Fig. 2a). Immunohistochemical staining revealed that inclusion bodies were positive for p62 and ubiquitin staining (Fig. 2b and c). Genetic testing revealed the patient was not a carrier of the fragile X mental retardation 1 (*FMR1*) premutation. Genetic examination was performed using repeat-primed PCR (RP-PCR) and GC-rich PCR (GC-PCR). We observed 102 GGC repeats in the 5’UTR of *NOTCH2NLC* (the normal range is < 40 [15]) (Fig. 2d, e).

During this hospitalization, the patient was given symptomatic treatment including an anti-antipsychotic (olanzapine 10 mg/daily) and nutrition support therapy, after which, her symptoms were relieved within 3 weeks. After discharge, a telephone follow-up at 3 months revealed the patient no longer had limb paralysis and mental hemiplegia as the first symptoms, and also fever, cognitive impairment and mental irritability from encephalitic attacks.

## Discussion and conclusions

In previous studies, Lin et al. reported a NIID patient with stroke-like onset symptoms, such as sudden limb weakness, and Li et al. reported a patient with multiple reversible encephalitic attacks, with light-headed episodes, central facial paralysis, unstable gait, aphasia, nausea, vomiting and loss of consciousness [16, 17]. However, no cases have yet been reported with stroke-like onset symptoms and encephalitic attacks. In this study, we reported such an NIID case with sudden aphasia and limb hemiplegia as the first symptoms, and also fever, cognitive impairment and mental irritability from encephalitic attacks.

A cranial MRI of NIID typically shows the following characteristic manifestations: (i) A DWI shows the curvilinear high signal of the cortex and medulla junction are specific [18]. The lesions progress from the bilateral frontal lobe to the parietal occipital lobe. The coexistence of U-shaped fiber linear high signals and decreased cognitive function in the DWI cortex are considered characteristic manifestations of NIID [19]. (ii) As NIID progresses, the T2/FLAIR high signal gradually affects the entire corpus callosum. It has been speculated that the range of abnormal signals at the corpus callosum is correlated to NIID progression [19]. (iii) NIID is often associated with brain atrophy and white matter lesions, but this is not specific.

Since 2003, several studies have reported eosinophilic nuclear inclusions in skin biopsies from NIID patients [12, 13, 20, 21], therefore skin biopsies have become useful in confirming NIID diagnoses. These biopsies revealed that inclusions were circular, with perinuclear diameters of 1.5–10 μm. They were ubiquitin and p62-positive, and when viewed under electron microscopy, they consisted of fibrous materials lacking membranous structures. However, it remains unclear what role p62 may play in the pathogenesis of NIID. As a ubiquitin-binding protein, p62 has important roles in several neurodegenerative diseases, but its significance in NIID remains as yet unclear [22].

GGC repeat expansion in the 5′ UTR of the *NOTCH2NLC* gene is associated with NIID mechanisms [10, 23]. Studies by Sone et al. and Tian et al. have suggested that the abnormal amplification of GGC trinucleotides in NIID, provides new molecular diagnostics for an NIID genetic diagnosis [10, 15]. Deng et al. [11] revealed that the abnormal expression of *NOTCH2NLC* could occur in both juvenile and adult patients with NIID. Equally, fragile X-related tremor/ataxia syndrome (FXTAS) also displays similar disease features during imaging, as the clinical symptoms of both conditions are similar [24]. In East Asia, NIID appears to be more common than FXTAS, thus before definitively diagnosing NIID, the *FMR1* gene must be analyzed to exclude FXTAS.

Our patient’s electrophysiological tests indicated that motor nerve conduction velocity of her limbs was slowed down. A Japanese study confirmed this in patients with NIID; these authors observed delays or reductions in the amplitude of nerve conduction velocity to varying degrees, similar to our observations [25]. Hirose et al. proposed that nerve conduction velocity and somatosensory evoked potentials may be the diagnostic basis for NIID [26].

Similar to other neurodegenerative diseases, no appropriate methods exist to definitively treat NIID pathophysiological mechanisms; only supportive therapies and symptomatic treatments are available. An NIID patient identified by Han et al., was given methylprednisolone pulse therapy (500 mg/daily) for three consecutive days, to gradually relived their symptoms [27]. In another Japanese case report, intravenous immunoglobulin (IVIg) therapy improved symptoms in a NIID patient [28]. Immunomodulatory therapy may be effective for NIID, but mechanisms must be explored in future studies. In this report, the patient’s mental irritability and cognitive impairment were improved by an anti-antipsychotic (olanzapine 10 mg/daily), suggesting disease relief may be related to the neuroprotective effects of olanzapine [29, 30].

According to our patient’s history, she suffered three episodes of limb weakness and two episodes of fever and aphasia, but she was not diagnosed due to a lack of radiographic examinations and genetic tests. We had also considered the possibility of central nervous system vasculitis or nervous system disease, but there was no evidence to support these diagnoses. The clinical heterogeneity of NIID at different stages of the disease increased the difficulty and complexity of our diagnosis.

In summary, we present a rare case of NIID with both stroke-like onset and encephalitic attacks. The patient was diagnosed based on characteristic DWI signals, intranuclear inclusions and expanded GGC repeats in NOTCH2NLC. Clinicians need to alert patients with recurrent disease stroke-like onset symptoms and/or encephalitic attack to avoid misdiagnosis.

## Data Availability

The datasets used and/or analyzed during this study are available from the corresponding author upon reasonable request.

## Abbreviations

NIID: Neuronal intranuclear inclusion disease
MRI: Magnetic resonance imaging
DWI: Diffusion-weighted imaging
CT: Cranial computed tomography
CTA: Computed tomography angiography
FMR1: Fragile X mental retardation 1
FXTAS: Fragile X-related tremor/ataxia syndrome
NOTCH2NLC: Notch 2 N-terminal like C
5′ UTR: 5′ untranslated region
RP-PCR: Repeat-primed PCR
GC-PCR: GC-rich PCR
IVIg: Intravenous immunoglobulin
EEG: Electroencephalogram
EEGs: Serial electroencephalogram

## References

[1] Sung J, Ramirez-Lassepas M, Mastri A, Larkin SM (1980). An unusual degenerative disorder of neurons associated with a novel intranuclear hyaline inclusion (neuronal intranuclear hyaline inclusion disease): a clinicopathological study of a case. Neuropathol Exp Neurol.

[2] Lindenberg R, Rubinstein L, Herman M, Haydon GB (1968). A light and electron microscopy study of an unusual widespread nuclear inclusion body disease. A possible residuum of an old herpesvirus infection. Acta Neuropathol.

[3] Kimber TE, Blumbergs PC, Rice JP, Hallpike JF, Edis R, Thompson PD, et al. Familial neuronal intranuclear inclusion disease with ubiquitin positive inclusions [J]. J Neurol Sci. 1998;160(1):33–40. 10.1016/S0022-510X(98)00169-5. 10.1016/s0022-510x(98)00169-5 9804114

[4] Pountney DL, Huang Y, Burns RJ, Haan E, Thompson PD, Blumbergs PC (2003). SUMO-1 marks the nuclear inclusions in familial neuronal intranuclear inclusion disease [J]. Exp Neurol.

[5] Mori F, Miki Y, Tanji K, Ogura E, Yagihashi N, Jensen PH, et al. Incipient intranuclear inclusion body disease in a 78-year-old woman [J]. Neuropathology. 2011;31(2):188–93. 10.1111/j.1440-1789.2010.01150.x. 10.1111/j.1440-1789.2010.01150.x 20667015

[6] Nakamura M, Murray ME, Lin WL, Kusaka H, Dickson DW (2014). Optineurin immunoreactivity in neuronal and glial intranuclear inclusions in adult-onset neuronal intranuclear inclusion disease [J]. Am J Neurodegener Dis.

[7] Mori F, Tanji K, Kon T, Odagiri S, Hattori M, Hoshikawa Y, et al. FUS immunoreactivity of neuronal and glial intranuclear inclusions in intranuclear inclusion body disease [J]. Neuropathol Appl Neurobiol. 2012;38:322–8. 10.1111/j.1365-2990.2011.01217.x. 10.1111/j.1365-2990.2011.01217.x 21883376

[8] Fiddes IT, Lodewijk GA, Mooring M, Bosworth CM, Ewing AD, Mantalas GL, et al. Human-specific NOTCH2NL genes affect NOTCH signaling and cortical neurogenesis [J]. Cell. 2018;173(6):1356–69. 10.1016/j.cell.2018.03.051. 10.1016/j.cell.2018.03.051 PMC5986104 29856954

[9] Suzuki IK, Gacquer D, Van Heurck R, Kumar D, Wojno M, Bilheu A, et al. Human-Specific NOTCH2NL Genes Expand Cortical Neurogenesis through Delta/Notch Regulation [J]. Cell. 2018;173:1370–1384.e16. 10.1016/j.cell.2018.03.067. 10.1016/j.cell.2018.03.067 PMC6092419 29856955

[10] Sone J, Mitsuhashi S, Fujita A, Mizuguchi T, Hamanaka K, Mori K, et al. Long-read sequencing identifies GGC repeat expansion in humanspecific NOTCH2NLC associated with neuronal intranuclear inclusion disease [J]. Nat Genet. 2019;51(8):1215–21. 10.1038/s41588-019-0459-y. 10.1038/s41588-019-0459-y 31332381

[11] Deng J, Gu M, Miao Y, Yao S, Zhu M, Fang P, et al. Long-read sequencing identified repeat expansions in the 5’-UTR of the gene from Chinese patients with neuronal intranuclear inclusion disease [J]. Med Genet. 2019;56(11):758–64. 10.1136/jmedgenet-2019-106268. 10.1136/jmedgenet-2019-106268 31413119

[12] Sone J, Kitagawa N, Sugawara E, Iguchi M, Nakamura R, Koike H, et al. Neuronal intranuclear inclusion disease cases with leukoencephalopathy diagnosed via skin biopsy [J]. J Neurol Neurosurg Psychiatry. 2014;85(3):354–6. 10.1136/jnnp-2013-306084. 10.1136/jnnp-2013-306084 24039026

[13] Takahashi-Fujigasaki J (2003). Neuronal intranuclear hyaline inclusion disease [J]. Neuropathology..

[14] Takahashi-Fujigasaki J, Nakano Y, Uchino A, Murayama S (2016). Adult-onset neuronal intranuclear hyaline inclusion disease is not rare in older adults [J]. Geriatr Gerontol Int.

[15] Tian Y, Wang J, Huang W, Zeng S, Jiao B, Liu Z (2019). Expansion of human-specific GGC repeat in neuronal intranuclear inclusion disease-related disorders. Am J Hum Genet.

[16] Lin P, Jin H, Yi K, He XS, Lin SF, Wu G (2020). A case report of sporadic adult neuronal intranuclear inclusion disease (NIID) with stroke-like onset [J]. Front Neurol.

[17] Li M, Li K, Li X, Tian Y, Shen L, Wu G, et al. Multiple reversible encephalitic attacks: a rare manifestation of neuronal intranuclear inclusion disease [J]. BMC Neurol. 2020;20(1):125. 10.1186/s12883-020-01712-5. 10.1186/s12883-020-01712-5 PMC7140360 32268889

[18] Yokoi S, Yasui K, Hasegawa Y, Niwa K, Noguchi Y, Tsuzuki T, et al. Pathological background of subcortical hyperintensities on diffffusion-weighted images in a case of neuronal intranuclear inclusion disease [J]. Clin Neuropathol. 2016;35(6):375–80. 10.5414/NP300961. 10.5414/NP300961 27719745

[19] Abe K, Fujita M. Over 10 years MRI observation of a patient with neuronal intranuclear inclusion disease [J]. BMJ Case Rep. 2017;2017:bcr2016218790. 10.1136/bcr-2016-218790. 10.1136/bcr-2016-218790 PMC5337643 28237949

[20] Sone J, Tanaka F, Koike H, Inukai A, Katsuno M, Yoshida M, et al. Skin biopsy is useful for the antemortem diagnosis of neuronal intranuclear inclusion disease [J]. Neurology. 2011;76(16):1372–6. 10.1212/WNL.0b013e3182166e13. 10.1212/WNL.0b013e3182166e13 21411744

[21] Sone J, Sobue G (2017). Neuronal intranuclear inclusion disease [J]. Brain Nerve.

[22] Bitto A, Lerner CA, Nacarelli T, Crowe E, Torres C, Sell C. P62/SQSTM1 at the interface of aging, autophagy, and disease [J]. Age (Dordr). 2014;36:9626. 10.1007/s11357-014-9626-3. 10.1007/s11357-014-9626-3 PMC4082582 24557832

[23] Ishiura H, Shibata S, Yoshimura J, Suzuki Y, Qu W, Doi K, et al. Noncoding CGG repeat expansions in neuronal intranuclear inclusion disease, oculopharyngodistal myopathy and an overlapping disease [J]. Nat Genet. 2019;51(8):1222–32. 10.1038/s41588-019-0458-z. 10.1038/s41588-019-0458-z 31332380

[24] Padilha IG, Nunes RH, Scortegagna FA, Pedroso JL, Marussi VH, Rodrigues Gonçalves MR, et al. MR imaging features of adult-onset neuronal intranuclear inclusion disease may be indistinguishable from fragile X-associated tremor/ataxia syndrome [J]. AJNR Am J Neuroradiol. 2018;39(9):E100–1. 10.3174/ajnr.A5729. 10.3174/ajnr.A5729 PMC7655280 30072371

[25] Sone J, Mori K, Inagaki T, Katsumata R, Takagi S, Yokoiet S (2016). Clinicopathological features of adult-onset neuronal intranuclear inclusion disease [J]. Brain..

[26] Hirose B, Hisahara S, Uesugi H, Sone J, Sobue G, Shimohama S (2018). Sporadic adult-onset neuronal intranuclear inclusion disease with abnormal electroretinogram, nerve conduction studies and somatosensory evoked potential [J]. Rinsho Shinkeigaku.

[27] Han X, Han M, Liu N, Xu J, Zhang Y, Zhang Y, et al. Adult-onset neuronal intranuclear inclusion disease presenting with typical MRI changes [J]. Brain Behav. 2019;9(12):e01477. 10.1002/brb3.1477. 10.1002/brb3.1477 PMC6908888 31749292

[28] Imai T, Kato B, Ohsima J, Hasegawa Y (2018). An adult onset sporadic neuronal intranuclear inclusion disease case reminiscent with fisher syndrome [J]. Rinsho Shinkeigaku.

[29] Chen AT, Nasrallah HA (2019). Neuroprotective effects of the second generation antipsychotics [J]. Schizophr Res.

[30] Xiong YJ, Song YZ, Zhu Y, Zuo WQ, Zhao YF, Shen X (2020). Neuroprotective effects of olanzapine against rotenone-induced toxicity in PC12 cells [J]. Acta Pharmacol Sin.

